# A minimally invasive, 3D-fluoroscopy-navigation-guided, 3D-controlled pedicle approach in spine surgery: first reliable results and impact on patient safety

**DOI:** 10.1007/s00068-020-01332-1

**Published:** 2020-03-02

**Authors:** André El Saman, Simon Lars Meier, Ingo Marzi

**Affiliations:** Department of Trauma, Hand and Reconstructive Surgery, University Hospital Frankfurt, Theodor-Stern-Kai 7, 60590 Frankfurt am Main, Germany

**Keywords:** Minimally invasive, MIS, 3D, Navigation, Pedicle screw, Spine surgery, Accuracy

## Abstract

**Purpose:**

Safe pedicle screw placement is a daily challenge to every spine surgeon. Introduction of minimally invasive approaches in spinal surgery led to an impaired facility of inspection of the surgical field increasing the importance of intraoperative imaging and navigation. During the past years, we established a minimally invasive, navigated approach in our clinical setting.

**Methods:**

We retrospectively reviewed the accuracy of pedicle approaches in patients treated due to traumatic or osteoporotic fractures, spondylitis/discitis, and tumoral lesions. Guide wires for pedicle screws or kyphoplasty cannulas were inserted in a 3D-navigation-guided, minimally invasive technique. Positioning of the guide wires was verified via 3D-scan, and pedicle screws/kyphoplasty cannulas were then visualized via a.p./lateral radiographs. Accuracy data were compared to a standard navigated open approach control group with indications similar to the MIS-group.

**Results:**

23 MIS patients were included in this study (25–84 years, mean 70 years) with a total of 154 placed guide wires. Handling of the navigated Jamshidi needle was easy and secure. The guide wires showed correct placement in 151/154 cases. Three wires (1.9%) needed correction of placement after control scan. There were no vascular or neurologic complications due to wire misplacement. In the open-surgery control group, 7/181 screws (3.9%) needed intraoperative correction presenting no significant difference compared to the correction rate of the MIS-group (*p* = 0.35).

**Conclusion:**

Our study shows the feasibility and reliability of a navigation-guided, minimally invasive pedicle approach in the clinical setting. Therefore, reduced morbidity due to minimized approaches can be combined with higher accuracy of navigated pedicle screw/kyphoplasty cannula placement improving patient safety.

## Introduction

Besides surgical expertise, technical progress and digitalization play an increasing role in spinal surgery. Indications for surgical procedures are extended to patients of increasing age with limited bone quality [1]. Several strategies were implemented during the past 20 years including kyphoplasty and use of fenestrated pedicle screws for cement augmentation to increase pullout strength in diminished bone density of the aging spine. While kyphoplasty is mainly used for osteoporotic fractures [2], an increasing number of fractures of the osteoporotic bone can be observed. This has even led to a new classification of spine fractures in the elderly [3]. In particular in these patients, a dedicated technique with minimal approaches to avoid soft-tissue problems is demanded. Thus, the surgical technique has to be adapted to allow a safe procedure and to minimize complications, such as wound healing or the requirement for revision.

Cement augmentation of pedicle screws can either be achieved with a cement application to the vertebral body prior to the insertion of the screw [4] or by use of fenestrated pedicle screws [5]. While design and number of holes in the fenestrated pedicle screw significantly influences the pullout strength and cement distribution, it must be assured that the screw fenestrations are securely placed in the vertebral body to reduce the risk of cement extrusion to the spinal canal [6–8]. Therefore, the accuracy of pedicle screw and kyphoplasty cannula placement is of utmost importance due to the risk for cement leakage that may result in compression of neural structures in the spinal canal if pedicle preparation failed to be exact.

Although having been controversially discussed in the past, computer-based navigation has proven itself as a valuable tool further enhancing precision in spinal surgery [9]. A recent metaanalysis by Meng et al. reported a lower malpositioning rate, a lower overall complication rate, and lower blood loss for patients who underwent computer-navigated thoracic pedicle screw placement [10]. The only major downside of computer navigation in spinal surgery seems to be the slightly increased operation duration [10].

We present a treatment concept including:

MIS, 3D-fluoroscopy navigation, 3D-accuracy control intraoperatively, and MIS cement augmentation if required.

In a series of patients, we combined 3D-fluoroscopy navigation with MIS-screw placement/kyphoplasty trocar placement and intraoperative 3D-scan. We determined the accuracy of intrapedicular guide wire placement and the need for intraoperative guide wire correction.

We hypothesized that although lacking tactile control after opening the pedicle, a 3D navigated MIS approach does not impair the accuracy of pedicle screw placement.

## Patients and methods

To elucidate the impact of MIS approach in 3D navigated spinal surgery, we performed a retrospective analysis of patient data from a single-level one trauma center. Patients who underwent spinal surgery either via navigated MIS or navigated open approach because of traumatic vertebral fractures, osteoporotic fractures, tumors, and infectious vertebral lesions were included in this study.

Data acquisition and processing was approved and performed in accordance with the regulations set forth by the Ethics Committee of the University Hospital Frankfurt (Frankfurt am Main, Germany; project no. 19-211) and in accordance with German law.

In the OR, we used a standardized surgical technique.

The patient was placed on the radiolucent carbon table in prone position. Prior to surgery, all metallic structures (i.e., cables, clips, etc.) were removed from the scanning field of the 3D-fluoroscopy device to avoid artifacts. The fractured vertebral body and the adjacent levels were marked on the patient’s skin.

After surgical disinfection and sterile draping, a skin incision of 2 cm in length was performed 2–3 levels distally of the injured vertebra. After hemostasis, a carbon reference clamp (Brainlab, Feldkirch, Germany) was fixed tight to the spinal process to avoid accidental loosening.

After relaxation and preoxygenation with inspiratory O_2_ concentration of 100%, a 3D-scan was recorded using high-resolution mode with 256 single images (Arcadis Orbic, Siemens, Erlangen, Germany). During data acquisition, the respirator was put on hold to avoid inaccuracy due to respiratory movement of the body.

The recorded data were then transferred to the navigation unit (Brainlab Vector Vision, Kolibri, Feldkirchen, Germany). With a navigated pointer, data accuracy was determined roughly comparing the match of actual anatomy (exposed spinal process as landmark) with the image on the navigation monitor as plausibility check.

**Fig. 1:**
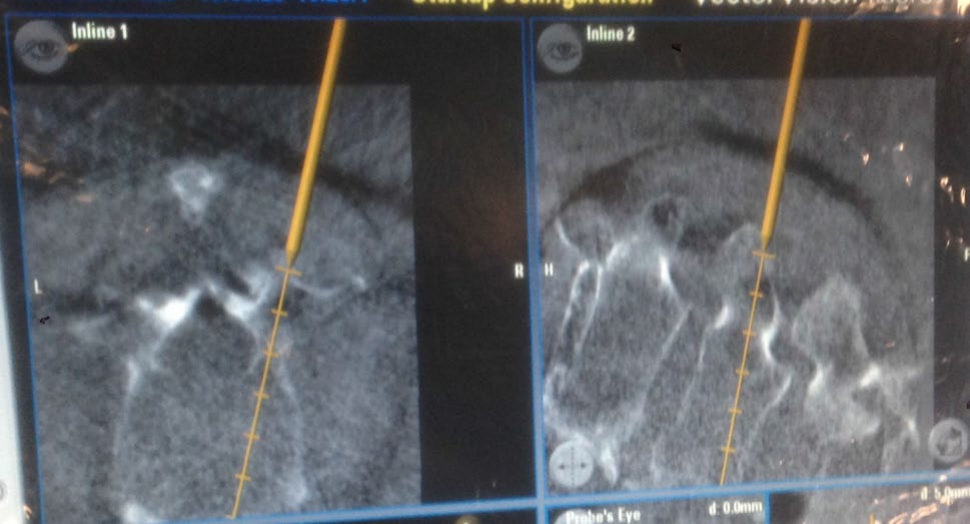
Trajectory of Jamshidi needle on navigation monitor

**Fig. 2:**
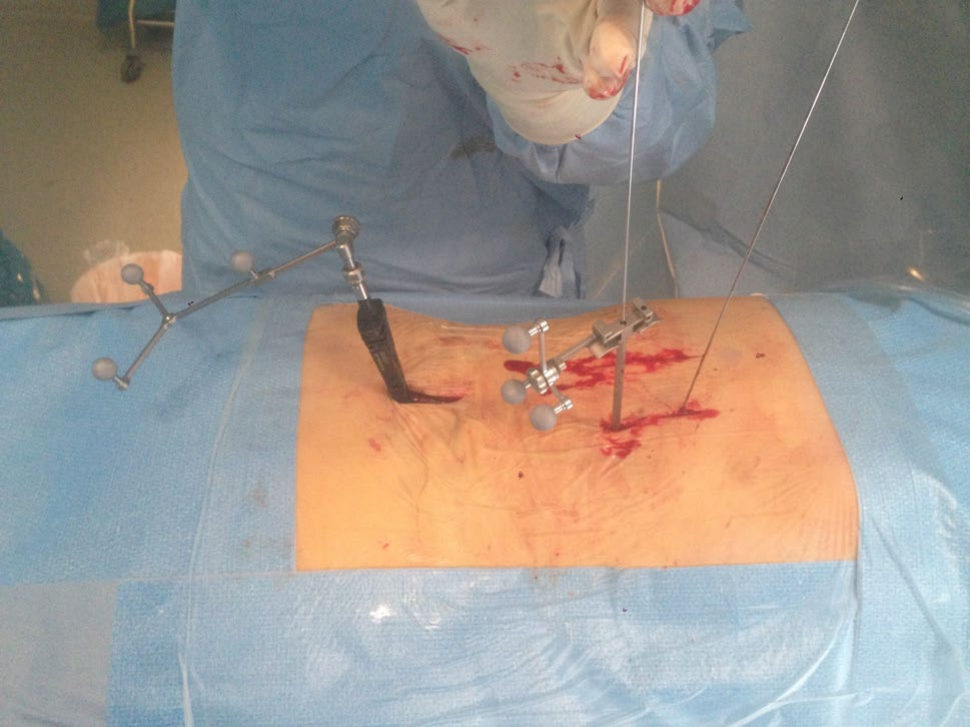
Insertion of guide wire via navigated Jamshidi needle

When the data set was judged reliable, region of skin incision for the pedicle screws was determined using a precalibrated, navigated 3.5 mm Jamshidi needle (Brainlab, Feldkirchen, Germany) with an inside trocar of 11G (Depuy Synthes, Umkirch, Germany). To locate the exact trajectory, we used a setting with a virtual offset of 50 mm (Fig. 1). After stab incision of 1.5 cm, the pedicle entry was approached using the navigated 3.5 mm Jamshidi needle and the pedicle was passed under navigation control. Via the transpedicular placed Jamshidi needle, a guide wire (2.0 mm/280 mm/1.45 mm, Depuy Synthes, Umkirch, Germany) was administered (Fig. 2).

K wires were used to guide either cannulated pedicle screws (Viper II //USS MIS, Depuy Synthes, Umkirch, Germany) or working cannulas for kyphoplasty (Depuy Synthes, Umkirch, Germany).

**Fig. 3:**
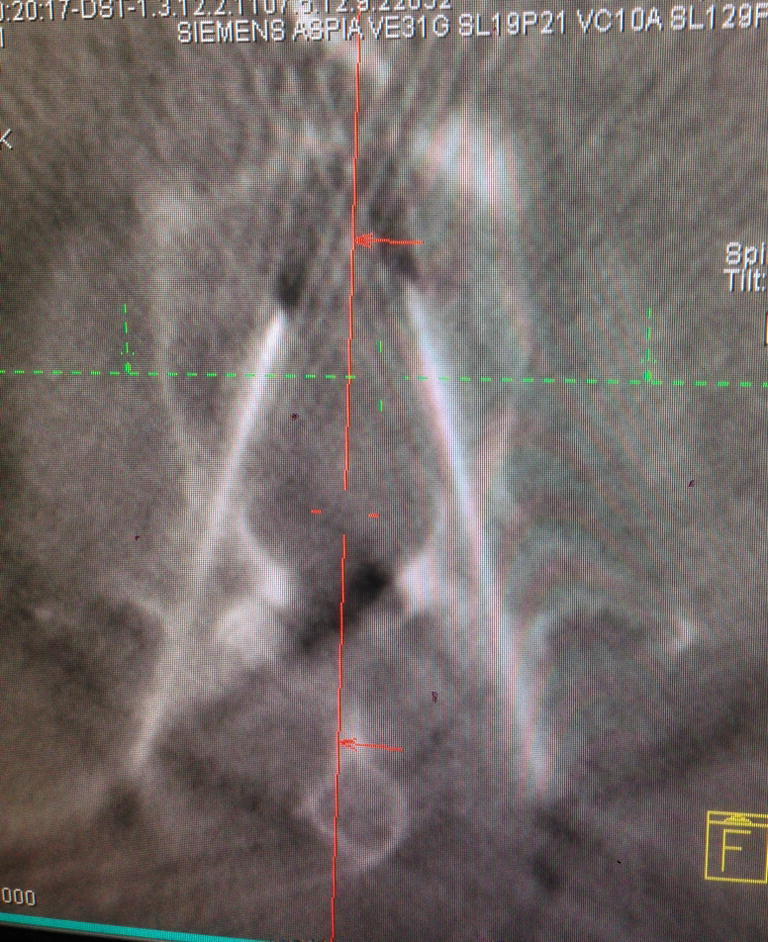
3D-Fluoroscopy control

Accuracy of the guide wire placement was determined by a subsequent 3D-scan (Fig. 3).

Data were postprocessed and analyzed in coronal, sagittal, parasagittal, and axial reconstructions. Special attention was paid to record violation of the pedicle walls medial, lateral, superior, and inferior. Any violation of the pedicle wall was recorded and the according guide wire was corrected, and any correction was judged as ‘misplacement’.

Then, length-adapted pedicle screws were administered using guide wires and a navigation controlled screw driver (Depuy Synthes, Umkirch, Germany).

In cases of transpedicular kyphoplasty without pedicle screw administration in the actual level, the pedicle was pertubated using a working sleeve, the vertebral body was prepared using plunger and ballon (Synflate, Depuy Synthes, Umkirch, Germany), the ballon was inflated to a means of 10 Atm, and the bone cement (Vertecem, Depuy-Synthes, Umkirch, Germany) was injected to a total amount of 2–3 ml per pedicle.

In cases using cement augmentation, PEEP was elevated during injection to prevent leakage.

Cement injection took place under fluoroscopy control in lateral view. Injection was stopped immediately when extrusion was detected.

The tactile control was followed by administration of a suitable pedicle screw (Matrix, DePuy Synthes, Umkirch, Germany; Omega, Zimmer-Biomet, Freiburg, Germany). After screw insertion, a 3D-scan (Arcadis Orbic, Siemens, Erlangen, Germany) was performed, and intrapedicular screw placement was analyzed. Screws with a violation of the pedicle wall > 2 mm were judged ‘misplaced’ and corrected during the same session.

In cases of kyphoplasty additional to posterior cement augmented instrumentation, the working cannulas and cement needles were left in place for approximately 18 min after injection until curing of vertecem was detected by slightly turning the needle within the working cannula. Posterior procedure was then completed by purging, hemostasis, and wound closure.

Data acquisition from the electronic patient record (Agfa HealthCare, Bonn, Germany) and intraoperative radiological findings from the 3D scans were processed using Microsoft Excel (Microsoft, Redmond, USA). Statistical analysis was performed using BiAS for Windows (Epsilon Verlag, Frankfurt/M., Germany) via two-sided Fisher’s exact test and Kruskal–Wallis-test, and the level of statistical significance was defined as *p* < 0.05.

## Results

23 MIS patients were included in this retrospective study. Traumatic vertebral fractures were the main cause for surgery (*n* = 16), followed by spondylodiscitis (*n* = 4), tumoral lesions (*n* = 2), and osteoporotic fractures (*n* = 1). Spinal instrumentation was carried out either bisegmental or multisegmental. One patient with morbid obesity was treated by navigation-guided, 3 D-controlled kyphoplasty alone of TH 12, L3, and L5 without instrumentation.

Epidemiologic data are shown in Table 1.

**Table 1:** Demographic and patient data overview

Patient	Age	Sex	Path. Level	Fracture	Tumor	Discitis	Class. AO	Class. OF	Ped. screws	Kyphoplasties	Bisegmental	Multisegmental	Cement	Misplacement	Cause	Miscellaneus
1	72	w	L1,L4	x			AO A3.3 2 x	OF 4 2 x	6	4		x	10	0	MVA	Spinal decompression
2	76	w	TH 11	x			AO B2.1	OF 5	7	0		x	0	1 (T12 left)	FALL	
3	72	w	TH12	x			AO A3.3	OF 4	4	2	x		6	0	FALL	
4	46	w	L3		x				4	0	x		0	0	TUMOR	
5	80	w	L3	x			AO A3.3	OF 4	4	2	x		6	0	FALL	
6	70	w	L5, L4, TH12	x			AO A3.1 3 x	OF 3 3 x	0	6		x	6	0	OF	Morbid obesity
7	48	w	L2, L4	x			AO A2.3/AO A2.1		6	6		x	6	0	FALL	Liver transplant listed
8	46	m	Th12, L1	x			AO B2.3		6	0		x	0	0	Fall/high energy	
9	25	w	L3	x			AO A3.1		4	0	x		0	0	MVA	
10	72	w	L1	x			AO A3.3	OF 4	8	0		x	8	0	Fall	
11	76	w	Th11/Th12			x			6	0		x	0	0	Discitis	Thoracoscopic discectomy, vancomycin + bone substitute
12	53	w	L1	x			AO A3,1		4	0	x		0	0	Fall	
13	51	m	L2	x			AO B2.3		4	0	x		0	0	MVA	Cage anterior
14	71	w	L1	x			AO A3.1	OF 3	8	0		x	0	0	Fall	
15	77	m	L3/L4			x			8	0		x	0	0	Discitis	Discectomy, vancomycin + bone substitute
16	62	m	L1	x			AO B2.3		4	2	x		6	0	Fall	
17	52	w	L1/2, L3/4			x			9	0		x	0	0	Discitis	Discectomy, vancomycin + bone substitute
18	84	w	Th9, TH11	x			AO A3.3	OF 4	8	0		x	4	0	Fall	
19	75	m	L2	x			AO B2.3	OF 4	8	0		x	4	1 (L3 left)	Fall	
20	37	m	L2	x			AO B1.2		4	0	x		0	1 (L1 left)	Fall	
21	62	m	L2/3			x			8	0		x	0	0	Discitis	Spinal decompression, discectomy, vancomycin + bone substitute
22	84	m	L2	x	x				8	0		x	6	0	Tumor	Spinal decompression
23	54	m	L2	x			AO A3.3		4	0	x		0	0	Fall/seizure	Cage anterior same procedure
	⌀ 62.8	14 w; 9m							∑ 132	∑ 22	∑ 9	∑ 14	∑ 62	∑ 3 (1.9%)		

Levels of pedicle approach ranged from TH 8 to L5. No cervical or sacral instrumentations were included in this analysis. Distribution of levels approached is shown in Table 2.

**Table 2:** Number of pedicle screws per level of instrumentation

Level of pedicular screw	< TH 8	TH8	TH9	TH10	TH11	TH12	L1	L2	L3	L4	L5	∑
MIS-group—number of screws administered	0	2	2	6	9	21	34	25	24	20	14	157
Open group—number of screws administered	48	4	8	20	24	20	24	15	12	6	0	181

Median age was 70 years, mean age 63 years (25–84), and male-to-female ratio was 9/14. A total of 154 pedicles were addressed using the 3D-fluoroscopy navigation-guided Jamshidi needle approach and the insertion of guide wires.

**Fig. 4:**
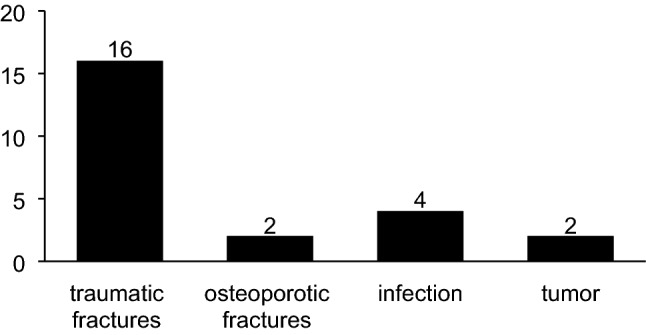
Indications for pedicle approach

Traumatic vertebral fractures led to the indication for surgery in the majority of cases (16/23), followed by spondylitis/spondylodiscitis (4/23), tumoral lesions (2/23), and osteoporotic fractures (1/23) (Fig. 4, Table 1).

Nine patients were treated by a limited bisegmental stabilization, in 14 individuals, a multisegmental approach was necessary, either due to limited bone quality or level of injury.

Cement augmentation techniques were used in *n* = 10 (43.5%) of patients (62/154, 40.5% of pedicle approaches), either as intravertebral kyphoplasty (22/154, 14.3%) or augmentation for fenestrated pedicle screws (40/154, 26%).

No cement was used in patients < 65 years of age with good bone quality, in cases of spondylodiscitis/spondylitis and in cases with very small pedicle diameters < 5 mm.

Distribution of levels approached with guide wires is shown in Table 2.

**Fig. 5:**
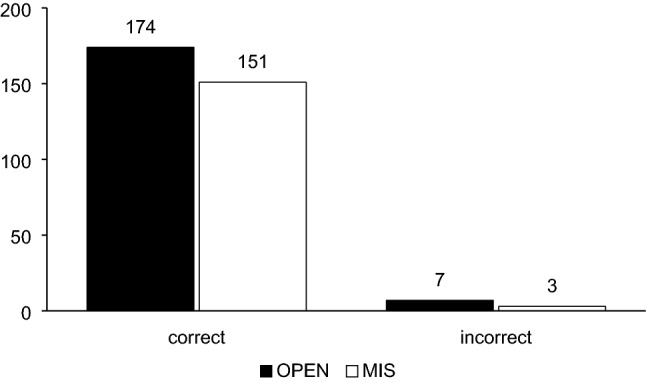
Results: Accuracy MIS vs open-surgery control group (*p* = 0.35)

In 154 pedicles, we saw a total of three wires with pedicle breach intraoperatively (1.9%, Fig. 5). All three inaccurate wires were located on the left side. We did not detect any correlation between level of injury and inaccuracy of pedicle instrumentation. We saw one inaccurate wire in TH 12, L1, and L3 each.

The misplaced wires were either corrected (2/3) or removed (1/3) prior to administration of screws. No revision surgery for inaccurate screws was needed. No patient suffered from neurologic or vascular complications. No cement leakage was detected. There was no statistical difference in accuracy of MIS procedures compared to our control group of open-surgery cases (Fig. 5, two-sided Fisher’s exact test, *p* = 0.35).

Procedure could be performed with 3D-navigation and 3D-control in all patients.

Additional anterior surgery was performed in five cases.

In cases of spondylodiscitis, anterior procedure [discectomy, lavage, and administration of local vancomycin and calcium sulphate hydroxyapatite-pellets (perOssal, aap, Berlin)] was performed after transfer to lateral position. In cases affecting the lumbar spine, we used an open retroperitoneal approach for anterior surgery. In one patient with spondylodiscitis affecting the Level T11/12 was treated via minimally invasive thoracoscopic approach. In one case of pincer-type fracture with affection of both vertebral endplates and adjacent discs (AO A3.2), we performed partial corporectomy and vertebral body replacement via an open retroperitoneal approach during the same surgery. One multiple trauma patient with AO B2.3 Fracture of L2 received additional anterior stabilization by vertebral body replacement 16 days after the initial posterior stabilization (Table 1).

Laminectomy was performed in three cases via a separate 3 cm midline incision. Decompression of the spinal canal was possible via this limited approach. These cases were included despite the “partial MIS” character of approach due to the technical similarity to the other MIS-cases.

Posterior surgery was then completed by minimally invasive construction of longitudinal rods (5.5 mm).

We identified 25 patients with a total of 181 pedicle screws for the navigated open control group. Twenty patients were treated with multisegmental instrumentation of four or more levels, and five patients received bisegmental instrumentation.

Indications for surgery in the control group were fractures of osteoporotic vertebral bodies (*n* = 10), fractures without osteoporosis (*n* = 5) and tumoral lesions (*n* = 10).

Mean age was 72 years (59–86).

Male-to-female ratio in the open-surgery group was 1:1.5. Levels of addressed vertebral pathologies ranged from T4 to L2 with a peak in the thoracolumbar region (TH 11–L1, *n* = 13). Pedicle screw approaches ranged from T2 to L4 (Table 2).

A total of seven screws showed a pedicle breach with violation of > 2 mm, were, therefore, judged as misplaced, and were corrected in the same session (7/181, 3.9%). No relevant violation of the vertebral bodies’ anterior cortex was detected. Levels of screw misplacement were L1 (*n* = 2), T12 (*n* = 2), T4 (*n* = 1), and L4 (*n* = 2). There was no preferation of any side (right: *n* = 3, left: *n* = 4).

In the control group, 12 patients received transfusion of at least one unit of packed red blood cells; 13 patients were treated without blood transfusion. In the MIS-group, 10 patients needed packed red blood cell transfusion; 13 patients showed no significant impairment of postoperative hemoglobin levels.

Due to the retrospective analysis of data, we were not able to collect reliable data concerning intraoperative blood loss by level of suction device collected blood.

Duration of surgery in the open navigated control group was slightly lower with a median value of 126 min (60–215 min); compared to the MIS study group (median 148 min, ranging 80–244 min), estimated time per screw was 15.2 min in the control group and 23 min in the MIS-group.

## Discussion

Minimally invasive procedures found widespread use in different surgical fields during the last 30 years. Main advantage is tissue preservation due to limited approach, leading to faster of mobility and reduced reconvalescence/hospital stay. Especially in spine surgery, limited muscular damage during posterior instrumentation showed to be beneficial concerning duration of inpatient treatment, time for recovery, analgesic dosage, and patient comfort [11, 12]. Reduced surgical morbidity, simplifying immediate postoperative care, and improving midterm functional results have been shown [13].

Accuracy of pedicle instrumentation plays an important role in spinal surgery. Besides the fear for iatrogenic neurological or vascular complications related to screw misplacement, another important aspect is lack of stability due to inaccurate intrapedicular and intravertebral fastening [14]. Recent reviews showed a superiority of navigation-guided techniques concerning screw accuracy compared to standard open surgery [15] with a preferation of 3D-fluoronavigation against the other techniques [16].

Eventually, the use of cement augmentation techniques requires absolute accuracy due to the danger of cement outflow to the spinal canal and subsequent compromise of spinal structures. Even in cases where screw positioning is perfect, we use the elevated PEEP technique to elevate intrathoracic pressure and to reduce the number of leakage complications [17]. Nevertheless, cement leakage can never be ruled out completely due to intravascular leakage via segmental or epidural vessels.

Gertzbein reported his experience with standard pedicle screw placement in 40 patients in 1990. Surgery was performed using lateral fluoroscopy and the AO internal fixator. Pedicle screw accuracy was then determined postoperatively by CT-scan. 81.5% of screws showed less than 2 mm encroachment of the medial border. Gertzbein postulated a safe zone of 4 mm medial to the pedicle border due to subarachnoid and epidural space. Any encroachment of the medial pedicle wall < 2 mm was considered irrelevant, 2–4 mm were considered acceptable, and > 4 mm was considered dangerous. His results led to the Gertzbein classification that is still used today [18], even if the majority of spine surgeons nowadays judge encroachment of < 2 mm as acceptable.

Zdichavsky developed another classification system, published in 2004. His classification is dependent on the portion of the pedicle screw diameter that is placed correctly, lateral or medial outside the pedicle or the vertebral body, respectively [19, 20]. This classification was based on a multicenter evaluation and showed a good intraobserver and interobserver reliability, but essential basic information, i.e., slice thickness of the different CT-scans performed was not provided. Other studies that used the Zdichavsky classification also fail to report the slice thickness of CT-scans performed. Another backdraft is the fact that compression of spinal structures by malpositioned screws is dependent on screw thickness and cannot be estimated by the classification of the screw alone (IIb classified pedicle screw is resulting in > 2 mm of obturation of the spinal canal in a 4 mm pedicle screw (which might be acceptable), but > 4 mm of compression of spinal structures in an 8 mm screw (which would rather be corrected).

Another classification published by Mirza et al. in 2003 sounds quite simple and reproducible: Grade 0 for intrapedicular screws without breaching, grade 1 < 2 mm perforation, grade 2 > 2 mm perforation, and grade 3 for total extrapedicular placement [21, 22].

Pedicle breaching of more than 2 mm is the basis for the majority of recent studies dealing with accuracy of pedicle screw placement [23, 24].

With the exception of intentional extrapedicular placement and taking into account another secure margin, the limit of 2 mm seems reliable and reproducible and was chosen as basis for our study concerning correct placement of the screws in the control group. (Mirza grade 1, Gertzbein grade 1). In the MIS-group, we accepted only guidewire placement with perfect intrapedicular placement to exclude any subsequent screw misplacement of > 2 mm according to the Gertzbein and Mirza classification.

The accuracy of navigation-guided pedicle screw placement techniques remains controversial. Bledsoe et al. reported a 100% rate of accuracy in a series of 150 pedicle screws in 2009 using fluoroscopy- and CT-guided navigation techniques [25]. Eck et al. reported a high rate of medial (10%) and lateral (56.7%) breaches in the thoracic spine using CT-navigated technique in 2013 [26]. In a series published by Ryang, there was a rate of correctly placed thoracic screws of 75.5% in a fluoroscopy-navigated series [27]. Miekisiak et al. found an accuracy of 79% in free-hand administered pedicle screws in a series leading to 2 revisions in 85 cases [28]. Kleck et al. published an accuracy of 94% in a CT-based navigation series using the O-arm CT-guided navigation technique distributed by Medtronic [29]. Kraus et al. saw an accuracy of 90.3% (< 2 mm breaching) in fluoroscopy navigation-guided pedicle screws compared to 94.6% in the standard technique pedicle screws in the thoracic and lumbar spine. They, therefore, postulated that computer-aided surgery does not improve the accuracy of dorsal pedicle screw placement [24].

Uehara et al. detected a perforation rate > 2 mm in 6.9% of screws in a CT-navigation-guided series including the cervical spine [23].

Compared to these studies, we saw a low perforation rate in our series, in particular in our MIS-cases (1.9% in the MIS-Group, 3.9% in the open-surgery control group).

Our approach combines the advantages of MIS technique with those of 3D-navigation-guided technique and intraoperative 3D-scan for the verification of intrapedicular placement. In our opinion, our technique has several benefits.

First of all, MIS surgery has an impact on muscular damage during surgery and, therefore, on duration of inpatient treatment, time for recovery, analgesic dosage, and patient comfort [11, 12].

Navigation-aided techniques have shown to improve pedicle screw accuracy in the previous studies. Combined with 3D-fluoroscopy control intraoperatively, surgical revision rate can be reduced obviously. We had no case of revision surgery in the present series, as we had in a series of open-surgery cases where we used intraoperative 3D-control, as well. Therefore, all pedicle approaches that show inacceptable pedicle wall violation can be corrected within the same surgery. In our series, none of the three patients with intraoperative correction of a misplaced guide wire suffered from neurologic or vascular damage.

A major backdraft of the MIS technique is the missing option for tactile control of the correct intrapedicular way using a simple probe, as we absolutely recommend in open-surgery procedures to detect inaccuracy of the virtual technique prior to pedicle instrumentation. We were anxious that this lack of palpation might lead to a higher rate of pedicle wall impairment in fluoroscopy navigation-guided MIS-cases. The actual data show that MIS pedicle approach is not less safe than open surgery, and there is even a tendency to higher accuracy. The effect of safe wire guidance of the navigated screw driver overrides the advantage of missing tactile control. Usage of the navigated Jamshidi needle technique for guide wire placement proved to be safe and secure in our study.

In our series, we performed the 3D-control after administration of the guide wires. This bears several advantages: first, the diameter of the K-wire is 1.45–2 mm, which is administered through the 3.5 mm Jamshidi needle. It is evident that there is less danger for severe damage of neural structures compared to pedicle screws with a diameter of up to 7 mm put in place before scanning to check for correct placement.

Second, the metal artifacts on the fluoroscopy pictures arising from the thin K wires are less extensive than those of pedicle screws with mounted connectors. Therefore, judgment of intrapedicular placement is more reliable.

In 40.5% of pedicle approaches, cement augmentation techniques, either for augmentation of screws to improve pullout strength, or for kyphoplasty were used. To our knowledge, this is the first description of MIS-, 3D-fluoroscopy-navigation-guided, and 3D-controlled cemented spinal surgery techniques so far. This is of special interest, because pedicle breaches in cement-aided spinal surgery techniques bear a special risk of cement extrusion to the spinal canal and to iatrogenic neurological complications.

In our clinical experience, in open surgery as well in MIS surgery, we make sure that any screw that is going to be cement augmented has to be checked by 3D-fluoroscopy for correct placement. If there is a pedicle breach detected on either side, no cement will be administered to this level.

In our study, we saw a malposition and need for correction in only 1.9% of pedicle approaches (3/154). 98.1% of accurate pedicle approaches is an excellent result of our series compared to the previous studies with rates ranging from 78 to 100% in open and MIS surgical procedures [14, 24–27, 29–31]. The fact that an additional haptic control of intrapedicular course of the implant is not possible in MIS surgery did not have any effect on accuracy in our series. The accuracy of the MIS cohort did not show a statistical significant worse value, there was even a tendency towards higher accuracy in the MIS-group. This shows the proof of feasibility for the prescribed technique that has potential to further improve patient safety.

**Fig. 6:**
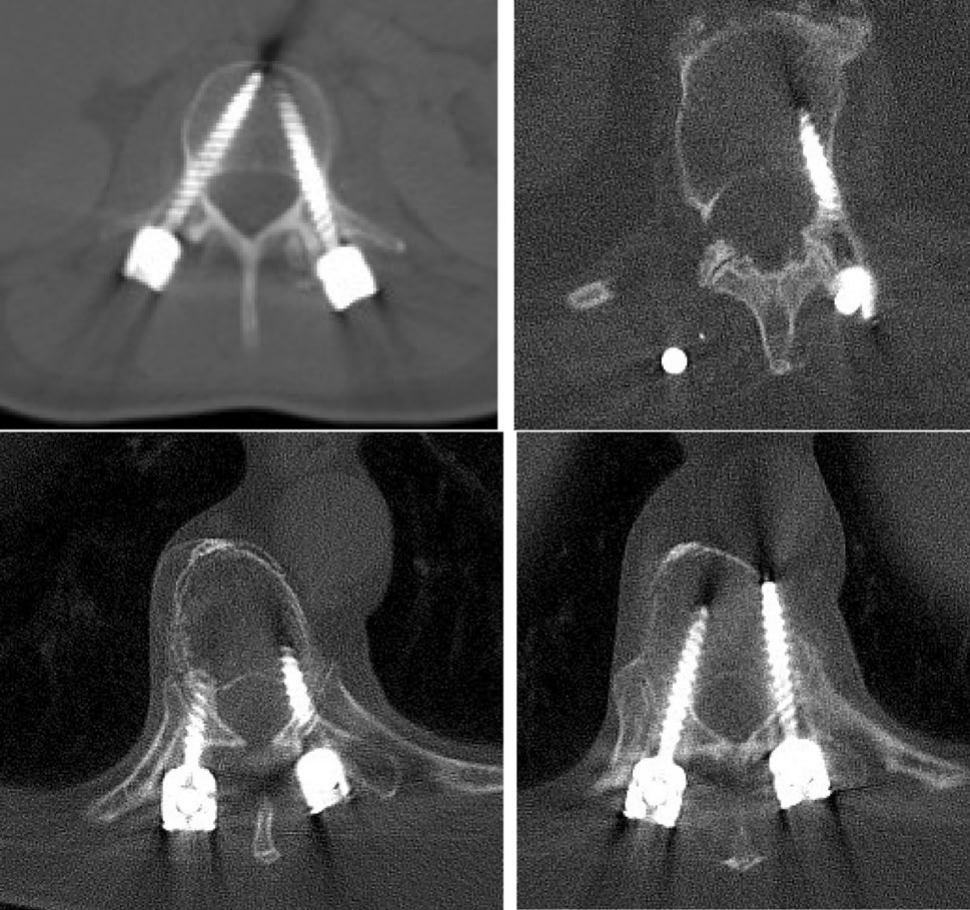
Screw accuracy postoperatively

We used our technique in a variety of different cases. Limited spinal decompression was possible as was instrumentation of scoliotic vertebral bodies with very small pedicles (Fig. 6). Limitations will occur if there is a major encroachment of the spinal canal leading to the necessity of a wide multisegmental laminectomy. Furthermore, we would not use the described technique if there is the need for extensive reposition maneuvers in type C fractures. In those cases, we prefer standard implants and open surgical approach.

In our study, we saw no difference between MIS and open groups concerning need for blood transfusion or time for surgery. We found a high number of patients in need for blood transfusion in both groups.

In our opinion, this is due to the fact that our groups are quite incoherent concerning indication for surgery, and, second, containing a high level of patients with tumoral lesions and preexisting anemia. Furthermore, a relevant number of patients received additional anterior stabilization in the same session and geriatric patients with preexisting cardiovascular risk factors were liberally transfused if hemoglobin level was < 8.5 mg/dl intra- or postoperative.

One patient in the MIS-group showed a blood loss > 2000 ml in posterior surgery due to severe liver cirrhosis despite limited approach. Indication in this case was a pincer-type fracture of T12 and L2 and successful spine surgery was mandatory prior to listing the patient for liver transplantation.

The additional tissue trauma for secure fixation of the reference clamp was very limited. Approach was performed via a 2 cm incision and a limited fascial incision of 1 cm on each side of the spinal process is sufficient for fixation and plausibility control by referencing the landmark with a navigated pointer. We did not notice any significant blood loss via this additional incision. There was no need for placement of draining tubes in any of the MIS-cases.

Recent studies showed a limited tissue damage and limitation of blood loss in cases of navigated spine surgery compared to open surgery [10]. Lee et al. even reported a shorter duration of the MIS surgical procedure compared to standard open technique for thoracolumbar and lumbar fractures [32].

In our series, we saw a trend towards prolonged surgical procedure time and towards reduced blood loss in the MIS-group, but there was no statistical significant difference. We have to state that our study group is quite inconsistent and, for example, need for additional laminectomy/decompression in both groups led to a limited value of “duration of surgery”.

Primum nihil nocere: This principle of avoiding iatrogenic damage for our patients is still a valuable guideline for surgeons. New strategies including the use of robotic assistance [33, 34] and intraoperative neuromonitoring [35, 36] were evaluated recently, leading to an inaccuracy rate between 0 and 7%. Further investigations will have to show the value of these additional techniques for patient outcome in spinal surgery procedures.

## Limitations of the present study

First of all, we are presenting retrospective data of a limited number of cases. Our results have to be verified by larger, prospective, controlled studies.

Our cohort is quite inconsistent concerning age and indications, but in our opinion, this fact proves the universal practicability of our approach not only in cases of traumatic and osteoporotic fractures but as well in cases of tumors and infections. Our trauma cases had a high percentage of AO-type B-injuries. Nevertheless, we saw a very low number of malpositioning with our technique, suggesting that fracture instability had no relevant effect on pedicle approach accuracy in the MIS-navigation setting.

## Conclusion

We present a complete approach to secure pedicle screw placement in a series of patients. Minimally invasive, 3D-fluoroscopy navigation-guided 3D-controlled pedicle instrumentation with or without cement augmentation techniques showed an accuracy of 98.1% in our series. We think that this is a valuable technique in selected patients. It will combine improvement of comfort (MIS, limited tissue impairment), stability (cement augmentation techniques), and safety (navigation-guided technique, intraoperative 3D-control).
